# Intensified ambulatory cardiology care: effects on mortality and hospitalisation—a comparative observational study

**DOI:** 10.1038/s41598-020-71770-9

**Published:** 2020-09-07

**Authors:** Olga A. Sawicki, Angelina Mueller, Anastasiya Glushan, Thorben Breitkreuz, Felix S. Wicke, Kateryna Karimova, Ferdinand M. Gerlach, Michel Wensing, Norbert Smetak, Ralph F. Bosch, Martin Beyer

**Affiliations:** 1 Institute of General Practice, Goethe University, Theodor-Stern-Kai 7, 60590 Frankfurt, Germany; 2 aQua, Institute for Applied Quality Improvement and Research in Health Care, 37073 Goettingen, Germany; 3 Department of General Practice and Health Services Research, University Hospital Heidelberg, Heidelberg, Germany; 4 Cardiology Practice, 73230 Kirchheim, Germany; 5 Cardio Centre Ludwigsburg-Bietigheim, 71634 Ludwigsburg, Germany

**Keywords:** Cardiology, Health care

## Abstract

Since 2010, an intensified ambulatory cardiology care programme has been implemented in southern Germany. To improve patient management, the structure of cardiac disease management was improved, guideline-recommended care was supported, new ambulatory medical services and a morbidity-adapted reimbursement system were set up. Our aim was to determine the effects of this programme on the mortality and hospitalisation of enrolled patients with cardiac disorders. We conducted a comparative observational study in 2015 and 2016, based on insurance claims data. Overall, 13,404 enrolled patients with chronic heart failure (CHF) and 19,537 with coronary artery disease (CAD) were compared, respectively, to 8,776 and 16,696 patients that were receiving usual ambulatory cardiology care. Compared to the control group, patients enrolled in the programme had lower mortality (Hazard Ratio: 0.84; 95% CI: 0.77–0.91) and fewer all-cause hospitalisations (Rate Ratio: 0.94; 95% CI: 0.90–0.97). CHF-related hospitalisations in patients with CHF were also reduced (Rate Ratio: 0.76; 95% CI: 0.69–0.84). CAD patients showed a similar reduction in mortality rates (Hazard Ratio: 0.81; 95% CI: 0.76–0.88) and all-cause hospitalisation (Rate Ratio: 0.94; 95% CI: 0.91–0.97), but there was no effect on CAD-related hospitalisation. We conclude that intensified ambulatory care reduced mortality and hospitalisation in cardiology patients.

## Introduction

In high income countries, cardiovascular disease (CVD), despite multiple drug- and device-related therapies and improvements in health-related life-styles, remains one of the leading causes of death and morbidity^1^. It is estimated that CVD is responsible for an annual 3.9 million deaths in Europe^2^. Its impact on patients, populations, and health systems is huge and is the cause of substantial healthcare utilization and cost, most of which is due to hospitalisation^3^. Furthermore, population ageing is likely to increase the prevalence and impact of CVD. Patients with cardiac diseases frequently consult both general practitioners (GPs) and cardiologists^4^. However, preventable mortality and morbidity often result from poor inter-sectoral coordination^5^. Although care coordination has been shown to optimise disease management, and improve patient outcomes^6,7^, notably in ambulatory care-sensitive conditions such as heart failure (CHF)^8^ and coronary artery disease (CAD)^9^, it is a major challenge to improve it within the framework of modern health systems^7^.

In Germany, the introduction of such programmes has been facilitated by the creation of a legal framework aimed at improving patient management in ambulatory care. In the past decade, ambulatory cardiology care, referred to here as the cardiology care programme, has been intensified by establishing voluntary contractual agreements between healthcare insurers, cardiologists, and GPs. The programme follows the stepped-care approach and reflects a renewed focus on GP-centred care^10–12^. Core features comprise elements of managed care^13^, including the regulation of healthcare provision, selective contracting with healthcare providers, and improved access to healthcare for cardiology patients^14^. Other elements of the programme (Table 1) include the promotion of guideline-recommended care, adherence to quality requirements, continuous quality improvement, participation in peer group training sessions, and the use of care pathways to coordinate care. Communication between GPs and cardiologists participating in the programme is standardised and requires the mutual exchange of important clinical information. The objective of this study was to assess the effect of this cardiology care programme on mortality and hospitalisation in enrolled patients with heart failure and coronary artery disease.

**Table 1:** Components of the cardiology care programme.

Components of the cardiology care programme
Structured disease management
Promotion of guideline-recommended care
New ambulatory medical services e.g., electrical cardioversion, specially trained healthcare assistants
Morbidity-adapted reimbursement
Incentives for repeat consultations in critical clinical situations and evidence-based pharmacotherapy
Adherence to quality requirements e.g. in diagnostics, a minimum of 100 echocardiograms must be carried out per quarter
Continuous data-driven quality improvement
Participation in clinical peer group training sessions e.g. in drug therapy
Coordinated care pathways with standardised communication between general practitioners and cardiologists
Patient education and emphasis on nationwide disease management programmes
Appointments for regular referrals within two weeks, and urgent referrals the same day

## Results

### Baseline characteristics

Of the 43,712 patients included in this study, 58.3% were male and 41.7% female. Mean age was 72.16 (SD 10.67) years, with a high proportion of hypertensive (89.2%), hyperlipidaemic (65.8%), and diabetic patients (41.2%). CHF patients were more likely to suffer from atrial fibrillation than CAD patients. In contrast, CAD patients were more likely to have a history of myocardial infarction. Overall, 13,404 patients with CHF and 19,537 with CAD enrolled in the intensified cardiology care programme, and were compared, respectively, to 8,776 and 16,696 control patients that were receiving usual ambulatory cardiology care. Table 2 displays baseline characteristics by disease cohort. The NYHA class, and Charlson comorbidity index score of patients in the intervention group were higher (3.95; SD 2.50) than those in the control group (3.48; SD 2.53).

**Table 2:** Baseline characteristics of patients by disease cohort.

Variables	CHF			CAD		
	Intervention group	Control group	*p* value	Intervention group	Control group	*p* value
Number of patients	13,404	8,776	n.a	19,537	16,696	n.a
Sociodemographic parameters						
Mean age (years)	72.9 ± 10.3	74.2 ± 11.4	< 0.0001	72.0 ± 9.9	72.1 ± 10.9	0.168
Age 18–40, n (%)	76 (0.6)	77 (0.9)		50 (0.3)	79 (0.5)	
Age 41–50, n (%)	372 (2.8)	246 (2.8)		550 (2.8)	578 (3.5)	
Age 51–60, n (%)	1,264 (9.4)	800 (9.1)		2,178 (11.1)	2,017 (12.1)	
Age 61–70, n (%)	2,723 (20.3)	1,495 (17.0)		4,551 (23.3)	3,543 (21.2)	
Age 71–80, n (%)	5,949 (44.4)	3,417 (38.9)		8,572 (43.9)	6,786 (40.6)	
Age 81–90, n (%)	2,855 (21.3)	2,438 (27.8)		3,483 (17.8)	3,388 (20.3)	
Age ≥ 91, n (%)	165 (1.2)	303 (3.5)		153 (0.8)	305 (1.8)	
Sex (% women)	44.8	47.2	< 0.0001	37.1	38.8	< 0.0001
German nationality (%)	91.5	90.9	0.118	89.1	88.5	0.067
Living in urban area (%)	46.5	47.2	0.313	48.9	49.0	0.769
Employed (%)	13.1	12.8	0.478	15.4	17.9	< 0.0001
Hardship status^a^ (%)	30.7	47.9	< 0.0001	29.4	40.6	< 0.0001
In need of nursing care (%)	11.6	23.0	< 0.0001	8.4	12.9	< 0.0001
Nursing home resident (%)	0.6	3.1	< 0.0001	0.3	1.3	< 0.0001
Health services utilisation (%)						
DMP CAD	41.7	22.7	< 0.0001	61.3	36.2	< 0.0001
DMP DM	36.8	25.7	< 0.0001	37.3	25.9	< 0.0001
CVD hospitalisation 2014	23.7	37.3	< 0.0001	21.3	27.6	< 0.0001
Influenza vaccination	49.3	41.7	< 0.0001	47.3	40.1	< 0.0001
Mean Charlson index score	4.6 ± 2.5	4.5 ± 2.6	< 0.003	4.0 ± 2.5	3.4 ± 2.6	< 0.0001
NYHA class (%)						
I/unknown	36.1	47.5	< 0.0001	63.8	79.8	< 0.0001
II	30.1	18.5		16.9	7.0	
III/IV	23.7	34.1		19.3	13.2	
Comorbid condition (%)						
Diabetes mellitus	44.4	43.0	0.035	44.3	40.4	< 0.0001
Hyperlipidaemia	65.7	60.2	< 0.0001	71.1	68.0	< 0.0001
Renal failure	25.8	31.3	< 0.0001	20.8	20.0	0.036
COPD	20.3	21.5	0.024	18.1	16.6	< 0.0001
Pneumonia in 2014	5.5	9.7	< 0.0001	4.2	5.2	< 0.0001
Depression	23.6	23.4	0.820	22.7	21.5	0.006
Cardiovascular history (%)						
Hypertension	91.8	90.0	< 0.0001	91.2	88.1	< 0.0001
CHF	100	100	n.a	47.0	33.2	< 0.0001
CAD	68.4	63.0	< 0.0001	100	100	n.a
Atrial fibrillation	36.8	42.1	< 0.0001	26.6	26.0	0.144
Other arrhythmias	50.4	43.3	< 0.0001	37.8	31.7	< 0.0001
Valvular heart disease	48.7	40.1	< 0.0001	37.1	29.0	< 0.0001
Myocardial infarction	24.4	21.4	< 0.0001	32.0	29.4	< 0.0001

Continuous variables are expressed as mean ± one SD. Categorical variables are presented as relative frequencies.

*CAD* coronary artery disease,* CHF* chronic heart failure,* COPD* chronic obstructive pulmonary disease,* CVD* cardiovascular disease,* DMP* disease management programme,* DM* diabetes mellitus.

^a^By limiting co-payments, hardship status avoids imposing additional financial hardship on chronically ill patients.

### Care coordination

We assessed the frequency of visits to cardiologists on a quarterly basis for the years 2015 and 2016. The mean number of quarterly visits to a cardiologist was 3.75 in CHF patients and 3.28 in CAD patients, and almost all visits were coordinated (3.72 in CHF patients and 3.23 in CAD patients). In the control group, the mean number of quarterly visits to a cardiologist was 3.66 in CHF patients and 3.58 in CAD patients. There were noticeable differences in care coordination. Control patients’ consultations with a cardiologist less frequently followed a referral from a GP (mean number of coordinated visits 2.44 in CHF patients and 2.32 in CAD patients).

### All-cause mortality

**Figure 1:**
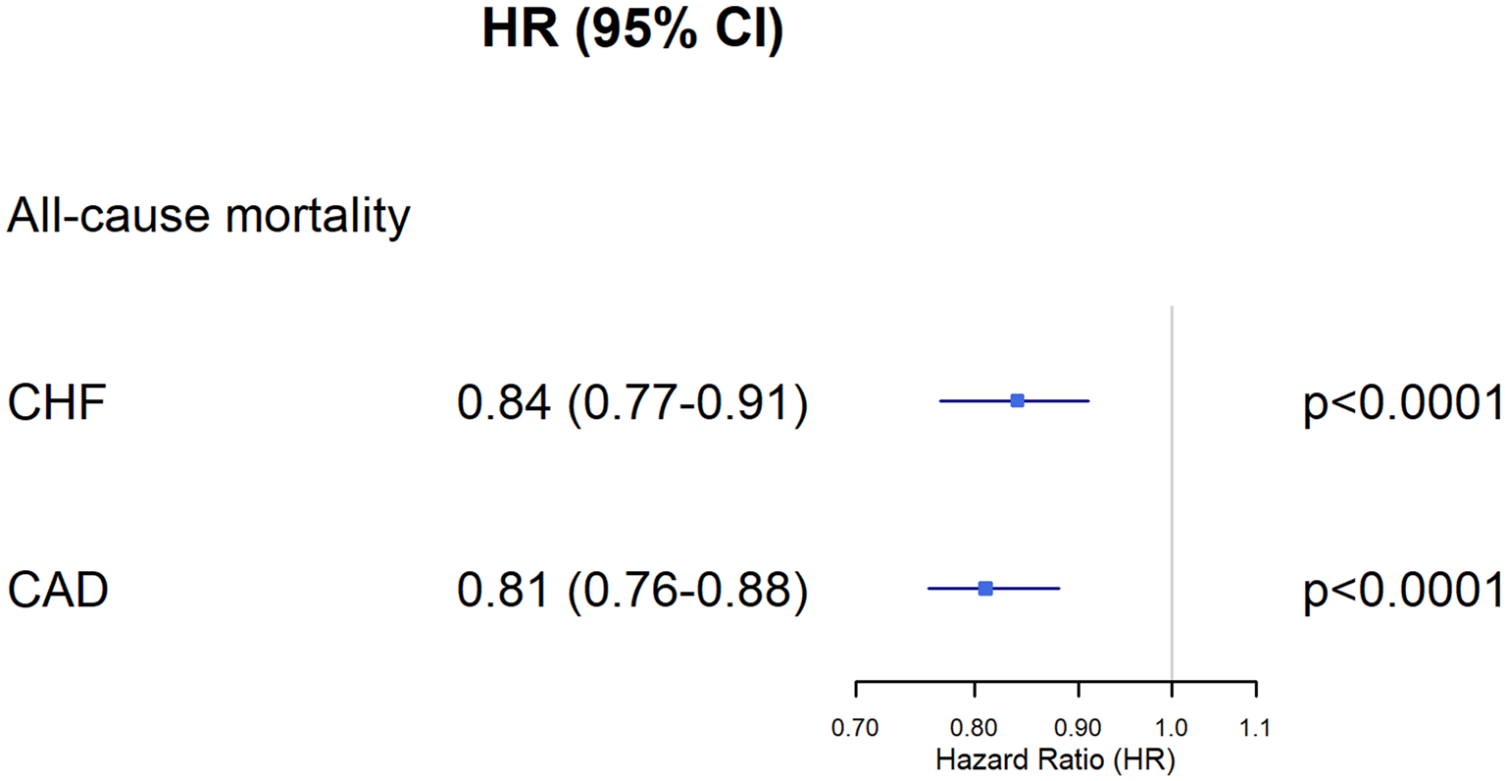
Forest plot of multivariable Cox regression models for all-cause mortality in CHF and CAD patients enrolled in the cardiology care programme versus control group. The squares and horizontal lines correspond to the hazard ratios and 95% confidence intervals. Hazard ratios are adjusted for baseline covariates including sociodemographic parameters, health services utilisation, mean Charlson index, NYHA class, comorbid conditions, and cardiovascular history as listed in Table 2. CAD, coronary artery disease; CHF, chronic heart failure; CI, confidence interval; HR, hazard ratio.

The results of the multivariable-adjusted model are presented in Fig. 1 and summarised online in Supplementary Tables S2 and S3. Compared to patients receiving usual care, intensified ambulatory care for CHF patients was associated with significantly lower all-cause mortality (Hazard Ratio 0.84; 95% CI 0.77–0.91; *p* < 0.0001) after adjusting for covariates. This effect was even larger in CAD patients, with a Hazard Ratio of 0.81 (95% CI 0.76–0.88; *p* < 0.0001). Men had increased risk of mortality as did older patients. As expected, a higher hazard ratio of death was associated with the presence of atrial fibrillation and greater Charlson index score.

### Hospitalisation

**Figure 2:**
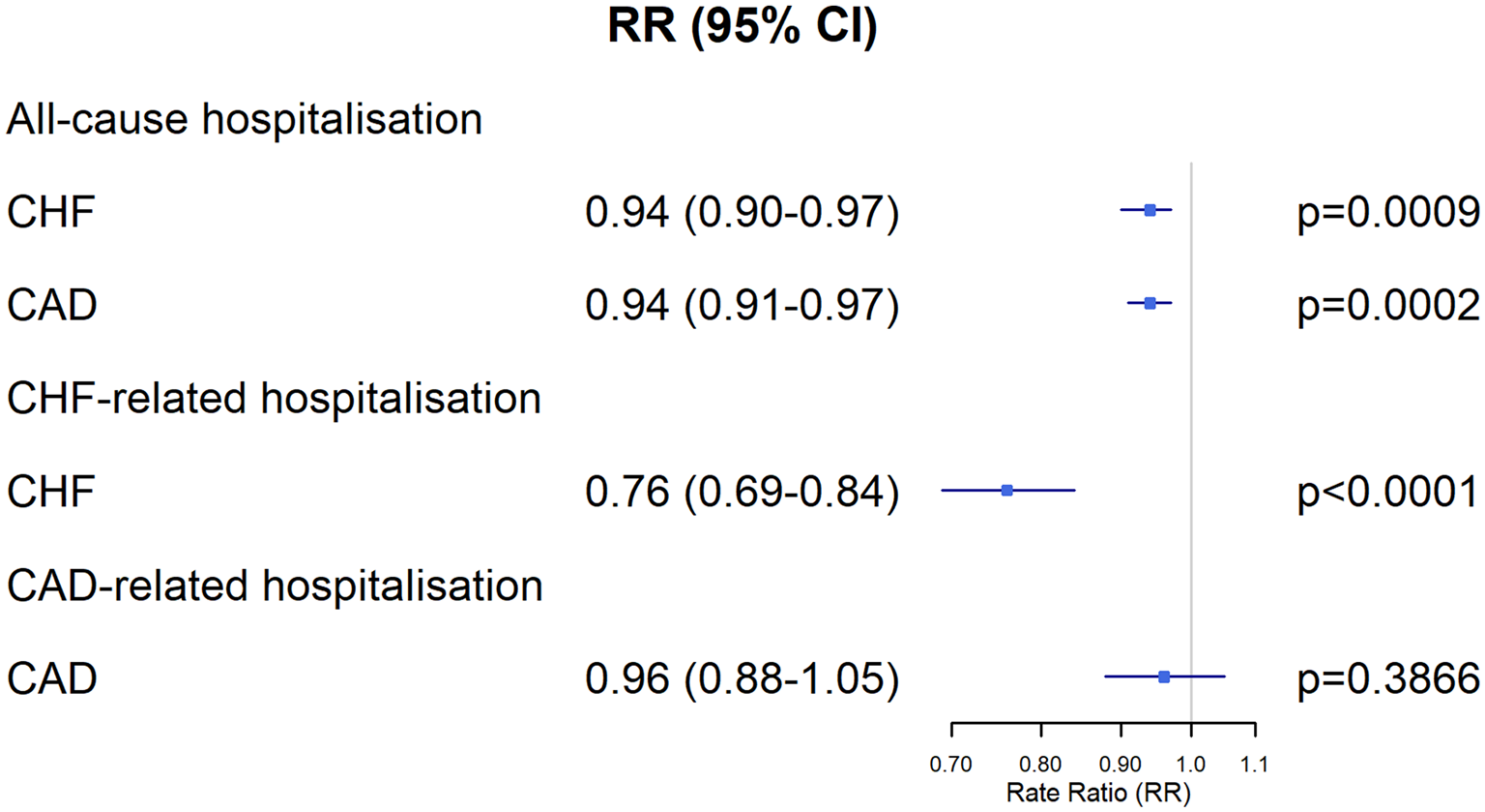
Forest plot of multivariable negative binomial regression models for hospitalisation in CHF and CAD patients enrolled in the cardiology care programme versus control group. The squares and horizontal lines correspond to the rate ratios and 95% confidence intervals. Rate ratios are adjusted for baseline covariates including sociodemographic parameters, health services utilisation, mean Charlson index, NYHA class, comorbid conditions, and cardiovascular history as listed in Table 2. CAD, coronary artery disease; CHF, chronic heart failure; CI, confidence interval; RR, rate ratio.

The results of the multivariable-adjusted regression models for hospitalisation rates are presented in Fig. 2 and summarised online in Supplementary Tables S4–S7. Compared to the control group, intensified ambulatory care in CHF patients was associated with fewer all-cause hospitalisations (RR 0.94; 95% CI 0.90–0.97; *p* = 0.0009), and CHF-related hospitalisations (Rate Ratio 0.76; 95% CI 0.69–0.84; *p* < 0.0001). For CAD patients, a similar pattern existed for all-cause hospitalisations with a corresponding adjusted Rate Ratio of 0.94 (95% CI 0.91–0.97; *p* = 0.0002). However, no association existed between the intervention and CAD-related hospitalisations in CAD patients (Rate Ratio 0.96; 95% CI 0.88–1.05; *p* = 0.3866). Presence of atrial fibrillation was consistently associated with an increased risk of hospitalisation. Influenza vaccination was protective, with the exception of CAD-related hospitalisation.

## Discussion

Ambulatory cardiology patients receive care from both GPs and cardiologists, but poor inter-sectoral coordination affects health outcomes. In this study of patients with two major cardiac disorders, we examined those that received intensified care provided collaboratively by a GP and a cardiac specialist as part of a comprehensive programme. We found that intensified ambulatory care reduces mortality and the likelihood of hospital admissions. Although the total number of visits to cardiologists was similar, patients receiving intensified cardiology care made more coordinated visits than those receiving usual care, which reflects the successful implementation of the programme. The fact that the cardiology care programme aims to structure care management, to increase guideline-oriented care, and to improve access^14^, likely contributed towards the reduction in mortality and hospitalisations. After adjusting for sociodemographic parameters, health service utilisation, and comorbidities, the risk of hospitalisation and mortality was clearly reduced in the intervention group compared with usual care. According to our covariates, we obtained results similar to those of other studies. Several studies have demonstrated an association between atrial fibrillation and increased risk of hospitalisation and mortality^15–17^. Pocock et al. identified age as the most powerful predictor of mortality and hospitalisation in CHF patients^18^.

The cardiology care programme is inherently multi-component. It is therefore impossible to determine the individual importance of each of the individual elements. However, the cardiology care programme employed many components of managed care that have been identified as contributing towards improving health outcomes^19,20^. Firstly, as the programme promotes evidence-based practice, and provides additional incentives for the use of evidence-based pharmacotherapy^14^, participating cardiologists were more likely to provide guideline-recommended care^21^. Secondly, several services were provided to outpatients for the first time, such as electrical cardioversion, pressure wire recording, implantation of cardioverter-defibrillators and loop recorders, which may have improved access to essential care^22^. Thirdly, as the programme incentivised repeat consultations in critical clinical situations, close monitoring may have resulted in earlier investigations and interventions^9^. The ambulatory monitoring strategy showed particular promise in detecting early warning signs before acute decompensation occurred in CHF patients^23^, hence preventing heart-failure-related readmissions^24^. Frequent patient-cardiologist interaction may also promote a variety of beneficial activities, such as preventive counselling, and nutritional assessments. Furthermore, as participation in the programme is linked to certain requirements such as the use of continuous data-driven quality improvement, participating cardiologists probably had greater expertise than those providing usual care. Process evaluation based on interviews with cardiologists^25^ suggests that cardiology practices in the programme initiated little change in their medical care^25^, with the interviewed cardiologists typically saying the quality of care they provided was already very good. The difference between intervention and control groups may therefore not result solely from the programme itself but partly reflect the selection of the ‘best physicians’ for participation. CAD-related hospitalisations were not reduced. However, since disease management programmes have been offered to CAD patients in southern Germany for more than a decade^26^, further improvements may be difficult to achieve.

Previous studies have focused on coordinated care in transitional care^8,9,27,28^. Particularly in high-risk patients with heart failure, randomized controlled studies have shown transitional care to be more clinically-effective and cost-effective than usual care^8,27^. Among these interventions, targeted care coordination was found to decrease hospitalisation and mortality^8,9,27^. The vast majority of coordinated care interventions focus on single-disease management^29^. The cardiology programme evaluated here, however, aims to provide high-quality healthcare delivery to a broader spectrum of ambulatory patients with varying cardiac disorders. We examined a wide range of important outcomes that are sensitive to ambulatory care for two major cardiac conditions. Our study provides useful insights into coordination between primary and secondary care, and the programme has demonstrated great promise in improving clinical and economic outcomes for a variety of cardiac conditions^21^. Models such as patient-centred medical homes^30^, and Accountable Care Organizations (ACO) in the Medicare Shared Savings Programme in the United States^31^ come closest to the cardiology care programme. But both accentuate primary care, without the deeper involvement of cardiologists^32^. Our results are consistent with the findings of ACO^33^.

Strengths of the study include its real-world population-based approach, sophisticated data analysis, and large sample size. The large sample size allowed us to adjust for many potential confounders. Use of claims data eliminated recall bias and allowed the programme to be evaluated objectively. However, the study was limited by our reliance on data (e.g., miscoding of NYHA classification). As data on cause of death were not available, it was not possible to conduct a more detailed analysis of the observed association of the programme with mortality. Furthermore, residual confounding from unmeasured confounders cannot be excluded. Finally, since participation in the cardiology contract is voluntary, we cannot exclude a self-selection bias by both patients and participating physicians. Although we used various covariates that we think will have controlled for selection bias, it is possible that a high number of very-low-risk patients and few high-risk patients were included in the programme. To ensure a representative sample of patients that actually require cardiac care, the selection of patients for both the control and intervention groups was based on patients that were being seen by a cardiologist. Regardless of participation in the programme, the overall quality of cardiovascular care in Baden-Wuerttemberg is high in comparison to other federal states in Germany^34^. In order to test the external validity of our results, demonstration projects of the coordinated care approach should be used in other German states, whereby their focus should also be on investigating ways of enhancing the sustainability of cardiology care.

## Methods

### Study design and participants

Based on routinely available claims data from the statutory health insurance fund ‘Allgemeine Ortskrankenkasse’ (AOK), a comparative observational study was conducted at the state level in Baden-Wuerttemberg, Germany, from 2015 to 2016. Baden-Wuerttemberg has about 11 million inhabitants^35^. AOK is the largest health insurance fund in this federal state, covering about 40% of the population at the time the study was conducted. About 70% of cardiologists and 15% of AOK-insured patients in Baden-Wuerttemberg participated in the cardiology programme^25^. Enrolled patients were compared with non-enrolled patients receiving usual care in ambulatory cardiology practices. We identified patients with diagnoses of CHF, CAD, or both, coded in accordance with the 10th Revision of the International Classification of Diseases. The two disease cohorts were created independently of one another and were not mutually exclusive. Eligible patients were ≥ 18 years of age, lived in Baden-Wuerttemberg, and were insured at the AOK without interruption during the observation period. Participation in nationwide disease management programmes for patients with chronic conditions (including diabetes, asthma/chronic obstructive pulmonary disease, and CAD) was not an exclusion criterion and was encouraged. Patients that were not seeing a cardiologist, or that switched groups during the observation period, were excluded (n = 260,897). Disease status and baseline characteristics of patients were recorded for a pre-observation period in 2014. Outcomes were assessed for a combination of the years 2015 and 2016.

The intervention group comprised eligible patients that had enrolled in the cardiology care programme and had seen a cardiologist that was a contract partner in the programme at least once during the pre-observation year 2014. The patients that were assigned to the control group had enrolled in neither the programme nor the GP-centred healthcare programme of the AOK, and had received usual care from a cardiologist that did not participate in the programme at least once during 2014. All patients enrolled in the programme gave their written informed consent before participation. Ethical approval was obtained from the local ethics committees of the Department of Medicine of Goethe-University in Frankfurt (No. 291/17). Reports on this observational study, which is part of an extensive evaluation report on collaborative care in Germany, were prepared in accordance with STROBE Statement and the German standard for secondary data analysis (STROSA)^36^. The evaluation has been registered at the German Clinical Trials Register (No. DRKS00014859).

### Cardiology care programme

The aim of the cardiology care programme is to manage chronic cardiac conditions, and improve health outcomes through intensified cardiology care and collaboration between healthcare providers. It is one of the medical specialist programmes that were developed as a part of a previously described special programme to enhance primary care (GP-centred healthcare programme)^10–12^ and only available to its participants. More than 1.6 million patients have enrolled in AOK Baden-Wuerttemberg’s GP-centred healthcare programme^21^. Enrolment in the specialist programme is voluntary for both patients and specialists. Enrolled patients only consult specialists that are contract partners in the programme, and specialist care requires referral by a GP (except for emergencies, gynaecologists, ophthalmologists, and paediatricians)^14^. This gate-keeping system aims to reduce the number of unnecessary specialist consultations.

The programme has its conceptual basis in the managed care approach to medicine. It combines structured medical care management with a renewed emphasis on guideline-recommended care^14^. It also makes new ambulatory medical services available, such as electrical cardioversion, pressure wire recording, implantation of cardioverter-defibrillators and loop recorders, as well as specially trained and certified healthcare assistants. Core elements of the programme for cardiologists are that physicians meet quality requirements, e.g. they base necessary adjustments on a data-driven improvement system, and participate regularly in clinical peer group training sessions involving GPs. Using a morbidity-adapted reimbursement system, additional incentives are available for repeat visits in various critical situations (e.g., cardiac decompensation), as well as evidence-based and cost-effective pharmacotherapy. Participating cardiologists work collaboratively with GPs in GP-centred healthcare programmes to generate individualised, disease-specific care pathways for patients, which take into consideration information such as medical history, medication lists, test results, and patient preferences. The fact that healthcare assistants work in concert with GPs and cardiologists likely contributed towards improving service coordination, including the transfer of information between providers. Regulations on documentation, clinical guideline adherence, and involvement of patients in the healthcare team promise to make care more comprehensive. Key features of the cardiology programme from the patient’s perspective include high continuity of care over time, patient education with an emphasis on nationwide disease management programmes, and prompt access to ambulatory cardiology care following GP-referral. Prompt access means a specialist appointment takes place within two weeks, or on the same day in urgent cases, and waiting times are shorter.

### Outcomes

The primary outcome in this study was all-cause mortality. For the cohort of CHF patients, secondary outcomes included all-cause, and CHF-related hospitalisations. Similarly, secondary outcomes for the CAD cohort were hospitalisation for all causes, and CAD-related hospitalisation. These outcome measures have been extensively validated in a previously published work^37^. We also documented frequency of visits to cardiologists on a quarterly basis, and coordinated contacts with cardiologists defined as cardiology consultations following GP referral. All measures were based on administrative data.

### Statistical analysis

For the purpose of data presentation, descriptive statistics were employed for the two disease cohorts. Between-group differences were statistically tested. Pearson’s chi-square test was used for categorical and binary variables (e.g. sex), and the t-test for continuous variables (e.g. age). Comparisons of outcomes in intervention and control groups were performed separately for each disease cohort. We applied multivariable analyses to deal with imbalances between the groups, and adjusted for differences in baseline variables (measured in year 1 of the observation period), i.e. sociodemographic parameters, health service utilisation, cardiovascular and non-cardiovascular comorbidities (Table 2). The 41 to 50 age-group was used as the reference category since the prevalence of cardiovascular risk factors in those over age 40 rises^38^. The Charlson index was used to summarize the burden of comorbidity^39^. The selection of variables for analysis was based on medical knowledge and availability in the administrative data, and they were defined in the same way as in previous evaluations (see Supplementary Table S1 online)^10,12^. No statistical criteria was used for selection of variables for the multivariable analysis. The negative binomial regression model and the Cox regression model were used to calculate, respectively, risk ratio for hospitalisation and hazard ratios for mortality. We tested for misspecification, e.g. the effects of separation and multicollinearity, in accordance with Good Practice in Secondary Data Analysis (GPS)^40^. Effects were expressed with 95% confidence intervals and two-tailed p-values. All descriptive and comparative analyses were carried out using SAS (version 9.4) and IBM SPSS Statistics (version 25).

## Supplementary information

Supplementary Information.

## Data Availability

AOK Baden-Wuerttemberg can be contacted for secondary analyses of their administrative data.
