# Supplementary material for: Intensified ambulatory cardiology care: effects on mortality and hospitalisation—a comparative observational study
Source: Sci Rep. 2020 Sep 7;10:14695. doi: 10.1038/s41598-020-71770-9 (PMC7477232; doi:10.1038/s41598-020-71770-9)
Supplement: Supplementary file 1 — Supplementary Information. [file 41598_2020_71770_MOESM1_ESM.pdf]

Intensified ambulatory cardiology care: effects on mortality and hospitalisation – a comparative observational study

Olga A. Sawicki[1,*], Angelina Mueller[1], Anastasiya Glushan[1], Thorben Breitkreuz[2], Felix S. Wicke[1], Kateryna Karimova[1], Ferdinand M. Gerlach[1], Michel Wensing[3], Norbert Smetak[4], Ralph F. Bosch[5,+] and Martin Beyer[1,+]

[1]Institute of General Practice, Goethe University, Theodor-Stern-Kai 7, Frankfurt, 60590, Germany
[2]aQua, Institute for Applied Quality Improvement and Research in Health Care, 37073 Goettingen, Germany

[3]Department of General Practice and Health Services Research, University Hospital Heidelberg, Germany

[4]Cardiology practice, Kirchheim, 73230, Germany
[5]Cardio Centre Ludwigsburg-Bietigheim, Ludwigsburg, 71634, Germany
[*]Corresponding author. Tel: + 49 69 6301 6476, Fax: + 49 69 6301 6428, E-mail address: sawicki@allgemeinmedizin.uni-frankfurt.de
[+]these authors contributed equally to this work

Supplementary Table S1. Definition of covariates

CAD, coronary artery disease; CHF, chronic heart failure; COPD, chronic obstructive pulmonary disease; CVD, cardiovascular disease; DMP, disease management programme; DM, diabetes mellitus.

**Covariate**	**Operationalisation**
Age	Age on January 1, 2015. Age-groups used for adjustment: 18-40, 41-50, 51-60, 61-70, 71-80, 81-90, ≥91
Sex	Male/female on January 1, 2015
German nationality	Yes/no on January 1, 2015
Living in urban area	Yes/no
Employed	Yes/no on January 1, 2015
State of hardship	Yes/no
In need of nursing care	Registered need for nursing care or no need of nursing care on January 1, 2015
Nursing home residence	Residing in nursing home or not residing in nursing home on January 1, 2015
DMP CAD	Yes/no
DMP DM	Yes/no
CVD hospitalisation	Yes/no. Hospitalisation in the year 2014 for any condition in ICD-10 chapter IX
Influenza vaccination	Yes/no. Influenza vaccination in quarters 3 or 4 of the year 2014 or quarter 1 of 2015 and in quarters 3 or 4 of the year 2015 or quarter 1 of 2016 respectively
Charlson index	Range, 0-37; based on diagnosed comorbidities in the year 2014, calculated according to Sundararajan et al.[34]
NYHA class	0 to IV of the New York Heart Association Grade of Heart Failure, based on ICD-10 codes in the year 2014. Used only for the CHF cohort
Comorbid condition	For all comorbid conditions: at least two outpatient diagnoses in two different quarters of one year, or one inpatient diagnosis, was required
Diabetes mellitus	ICD-10: E10, E11
Hyperlipidaemia	ICD-10: E78
Renal failure	ICD-10: N18.2, N18.3, N18.4, N18.5 , N18.8, N18.9
COPD	ICD-10: J44
Pneumonia	ICD-10: J12-J18, U69.00, A40.3, J86, B95.3
Depression	ICD-10: F32, F33
Hypertension	ICD-10: I10-I15
CHF	ICD-10: I50
CAD	ICD-10: I20-I25
Atrial fibrillation	ICD-10: I48
Other arrhythmias	ICD-10: I42.80, I44-I47, I49, Q24.6
Valvular heart disease	ICD-10: I05-I08, I34-I39, Q20-26
Myocardial infarction	ICD-10: I21, I22, I25.2

Supplementary Table S2. Multivariable-adjusted Cox regression models for all-cause mortality in CHF patients enrolled in the cardiology care programme versus control group

CAD, coronary artery disease; CHF, chronic heart failure; COPD, chronic obstructive pulmonary disease; CVD, cardiovascular disease; DMP, disease management programme; DM, diabetes mellitus.

Variables	Interpretation	Hazard Ratio	Lower 95% CI	Upper 95% CI	*p*-value
Group	Cardiology care programme versus control group	0.840	0.774	0.911	<0.0001
Age	18-40 versus 41-50 years	0.948	0.358	2.505	0.9135
Age	51-60 versus 41-50 years	0.991	0.617	1.594	0.9716
Age	61-70 versus 41-50 years	1.513	0.974	2.352	0.0656
Age	71-80 versus 41-50 years	2.023	1.310	3.124	0.0015
Age	81-90 versus 41-50 years	3.431	2.219	5.304	<0.0001
Age	91+ versus 41-50 years	5.351	3.393	8.440	<0.0001
Sex	Male versus female	1.567	1.446	1.698	<0.0001
German nationality	No versus yes	0.870	0.739	1.025	0.0960
Living in urban area	No versus yes	1.047	0.972	1.127	0.2245
Employed	Yes versus no	0.737	0.615	0.883	0.0009
State of hardship	Yes versus no	1.046	0.965	1.134	0.2758
In need of nursing care	Yes versus no	2.419	2.212	2.645	<0.0001
Nursing home resident	Yes versus no	1.670	1.426	1.956	<0.0001
DMP CAD	Yes versus no	0.933	0.849	1.026	0.1524
DMP DM	Yes versus no	1.009	0.905	1.125	0.8728
CVD hospitalisation	Yes versus no	1.070	0.980	1.168	0.1326
Influenza vaccination	No versus yes	1.137	1.056	1.225	0.0007
Charlson index	Per point	1.112	1.092	1.133	<0.0001
NYHA class	Class II versus I	0.974	0.806	1.176	0.7829
NYHA class	Class III versus I	1.456	1.217	1.741	<0.0001
NYHA class	Class IV versus I	1.852	1.527	2.246	<0.0001
NYHA class	No NYHA class versus class I	1.250	1.045	1.495	0.0148
Diabetes mellitus	Yes versus no	0.918	0.826	1.021	0.1154
Hyperlipidaemia	Yes versus no	0.848	0.783	0.918	<0.0001
Renal failure	Yes versus no	1.244	1.139	1.359	<0.0001
COPD	Yes versus no	1.079	0.992	1.175	0.0776
Pneumonia	Yes versus no	1.188	1.066	1.324	0.0018
Depression	Yes versus no	0.938	0.858	1.024	0.1530
Hypertension	Yes versus no	0.777	0.675	0.895	0.0005
CAD	Yes versus no	0.949	0.864	1.044	0.2815
Atrial fibrillation	Yes versus no	1.237	1.144	1.338	<0.0001
Other arrhythmias	Yes versus no	0.930	0.863	1.002	0.0566
Valvular heart disease	Yes versus no	1.112	1.031	1.199	0.0058
Myocardial infarction	Yes versus no	1.021	0.928	1.124	0.6721

Supplementary Table S3. Multivariable-adjusted Cox regression models for all-cause mortality in CAD patients enrolled in the cardiology care programme versus control group

CAD, coronary artery disease; CHF, chronic heart failure; COPD, chronic obstructive pulmonary disease; CVD, cardiovascular disease; DMP, disease management programme; DM, diabetes mellitus.

Variables	Interpretation	Hazard Ratio	Lower 95% CI	Upper 95% CI	*p*-value
Group	Cardiology care programme versus control group	0.814	0.756	0.878	<0.0001
Age	18-40 versus 41-50 years	1.300	0.388	4.360	0.6711
Age	51-60 versus 41-50 years	1.042	0.648	1.678	0.8645
Age	61-70 versus 41-50 years	1.787	1.142	2.796	0.0110
Age	71-80 versus 41-50 years	2.568	1.647	4.004	<0.0001
Age	81-90 versus 41-50 years	4.501	2.881	7.033	<0.0001
Age	91+ versus 41-50 years	8.729	5.471	13.93	<0.0001
Sex	Male versus female	1.596	1.476	1.725	<0.0001
German nationality	No versus yes	0.887	0.771	1.020	0.0929
Living in urban area	No versus yes	1.012	0.944	1.085	0.7398
Employed	Yes versus no	0.794	0.676	0.933	0.0049
State of hardship	Yes versus no	1.126	1.043	1.215	0.0024
In need of nursing care	Yes versus no	2.620	2.403	2.857	<0.0001
Nursing home resident	Yes versus no	1.547	1.290	1.856	<0.0001
DMP CAD	Yes versus no	0.863	0.799	0.933	0.0002
DMP DM	Yes versus no	0.999	0.901	1.108	0.9883
CVD hospitalisation	Yes versus no	1.069	0.986	1.158	0.1041
Influenza vaccination	No versus yes	1.164	1.085	1.250	<0.0001
Charlson index	Per point	1.141	1.121	1.161	<0.0001
Diabetes mellitus	Yes versus no	0.907	0.821	1.003	0.0567
Hyperlipidaemia	Yes versus no	0.867	0.803	0.936	0.0002
Renal failure	Yes versus no	1.222	1.122	1.330	<0.0001
COPD	Yes versus no	1.149	1.058	1.247	0.0010
Pneumonia	Yes versus no	1.249	1.117	1.397	<0.0001
Depression	Yes versus no	0.940	0.864	1.022	0.1492
Hypertension	Yes versus no	0.852	0.744	0.976	0.0208
CHF	Yes versus no	1.274	1.175	1.382	<0.0001
Atrial fibrillation	Yes versus no	1.294	1.200	1.396	<0.0001
Other arrhythmias	Yes versus no	0.973	0.905	1.047	0.4717
Valvular heart disease	Yes versus no	1.130	1.050	1.217	0.0011
Myocardial infarction	Yes versus no	1.044	0.967	1.128	0.2704

Supplementary Table S4. Multivariable-adjusted negative binomial regression models for all-cause hospitalisation in CHF patients enrolled in the cardiology care programme versus control group
CAD, coronary artery disease; CHF, chronic heart failure; COPD, chronic obstructive pulmonary disease; CVD, cardiovascular disease; DMP, disease management programme; DM, diabetes mellitus.

Variables	Interpretation	Rate Ratio	Lower 95% CI	Upper 95% CI	*p*-value
Group	Cardiology care programme versus control group	0.935	0.898	0.973	0.0009
Age	18-40 versus 41-50 years	0.982	0.758	1.272	0.8910
Age	51-60 versus 41-50 years	1.012	0.889	1.152	0.8572
Age	61-70 versus 41-50 years	1.023	0.902	1.160	0.7210
Age	71-80 versus 41-50 years	1.166	1.029	1.321	0.0158
Age	81-90 versus 41-50 years	1.260	1.109	1.432	0.0004
Age	91+ versus 41-50 years	1.186	0.990	1.421	0.0641
Sex	Male versus female	1.167	1.123	1.213	<0.0001
German nationality	No versus yes	0.916	0.858	0.978	0.0086
Living in urban area	No versus yes	1.012	0.976	1.049	0.5164
Employed	Yes versus no	0.879	0.823	0.938	0.0001
State of hardship	Yes versus no	1.556	1.496	1.617	<0.0001
In need of nursing care	Yes versus no	1.343	1.273	1.417	<0.0001
Nursing home resident	Yes versus no	0.991	0.850	1.155	0.9045
DMP CAD	Yes versus no	1.001	0.957	1.048	0.9503
DMP DM	Yes versus no	1.005	0.949	1.064	0.8706
CVD hospitalisation	Yes versus no	1.280	1.225	1.336	<0.0001
Influenza vaccination	No versus yes	1.038	1.001	1.077	0.0436
Charlson index	Per point	1.077	1.066	1.088	<0.0001
NYHA class	Class II versus I	1.013	0.944	1.087	0.7251
NYHA class	Class III versus I	1.224	1.140	1.315	<0.0001
NYHA class	Class IV versus I	1.405	1.288	1.533	<0.0001
NYHA class	No NYHA class versus class I	1.099	1.025	1.179	0.0081
Diabetes mellitus	Yes versus no	0.941	0.891	0.994	0.0307
Hyperlipidaemia	Yes versus no	0.937	0.901	0.975	0.0013
Renal failure	Yes versus no	1.178	1.125	1.235	<0.0001
COPD	Yes versus no	1.152	1.102	1.204	<0.0001
Pneumonia	Yes versus no	1.231	1.150	1.318	<0.0001
Depression	Yes versus no	1.049	1.005	1.094	0.0289
Hypertension	Yes versus no	1.001	0.936	1.071	0.9683
CAD	Yes versus no	1.035	0.987	1.084	0.1568
Atrial fibrillation	Yes versus no	1.227	1.181	1.275	<0.0001
Other arrhythmias	Yes versus no	0.988	0.952	1.025	0.5099
Valvular heart disease	Yes versus no	1.042	1.005	1.082	0.0266
Myocardial infarction	Yes versus no	1.038	0.990	1.089	0.1268

Supplementary Table S5. Multivariable-adjusted negative binomial regression models for all-cause hospitalisation in CAD patients enrolled in the cardiology care programme versus control group

CAD, coronary artery disease; CHF, chronic heart failure; COPD, chronic obstructive pulmonary disease; CVD, cardiovascular disease; DMP, disease management programme; DM, diabetes mellitus.

Variables	Interpretation	Rate Ratio	Lower 95% CI	Upper 95% CI	*p*-value
Group	Cardiology care programme versus control group	0.943	0.914	0.973	0.0002
Age	18-40 versus 41-50 years	1.394	1.069	1.818	0.0143
Age	51-60 versus 41-50 years	1.182	1.067	1.311	0.0014
Age	61-70 versus 41-50 years	1.220	1.102	1.350	0.0001
Age	71-80 versus 41-50 years	1.381	1.247	1.529	<0.0001
Age	81-90 versus 41-50 years	1.518	1.365	1.687	<0.0001
Age	91+ versus 41-50 years	1.809	1.530	2.138	<0.0001
Sex	Male versus female	1.153	1.117	1.190	<0.0001
German nationality	No versus yes	0.883	0.840	0.927	<0.0001
Living in urban area	No versus yes	1.012	0.983	1.042	0.4255
Employed	Yes versus no	0.951	0.904	1.000	0.0509
State of hardship	Yes versus no	1.602	1.552	1.654	<0.0001
In need of nursing care	Yes versus no	1.370	1.303	1.440	<0.0001
Nursing home resident	Yes versus no	0.865	0.726	1.032	0.1079
DMP CAD	Yes versus no	0.976	0.946	1.007	0.1329
DMP DM	Yes versus no	0.962	0.918	1.009	0.1142
CVD hospitalisation	Yes versus no	1.308	1.263	1.354	<0.0001
Influenza vaccination	No versus yes	1.077	1.045	1.110	<0.0001
Charlson index	Per point	1.095	1.085	1.104	<0.0001
Diabetes mellitus	Yes versus no	0.961	0.918	1.006	0.0899
Hyperlipidaemia	Yes versus no	0.986	0.954	1.019	0.3892
Renal failure	Yes versus no	1.131	1.086	1.179	<0.0001
COPD	Yes versus no	1.162	1.118	1.207	<0.0001
Pneumonia	Yes versus no	1.265	1.185	1.351	<0.0001
Depression	Yes versus no	1.091	1.053	1.130	<0.0001
Hypertension	Yes versus no	1.096	1.040	1.155	0.0007
CHF	Yes versus no	1.041	1.007	1.077	0.0168
Atrial fibrillation	Yes versus no	1.251	1.209	1.295	<0.0001
Other arrhythmias	Yes versus no	1.028	0.996	1.061	0.0881
Valvular heart disease	Yes versus no	1.059	1.025	1.093	0.0005
Myocardial infarction	Yes versus no	0.988	0.956	1.022	0.4962

Supplementary Table S6. Multivariable-adjusted negative binomial regression models for CHF-related hospitalisation in CHF patients enrolled in the cardiology care programme versus control group
CAD, coronary artery disease; CHF, chronic heart failure; COPD, chronic obstructive pulmonary disease; CVD, cardiovascular disease; DMP, disease management programme; DM, diabetes mellitus.

**Variables**	**Interpretation**	**Rate Ratio**	**Lower 95% CI**	**Upper 95% CI**	***p*-value**
Group	Cardiology care programme versus control group	0.761	0.688	0.841	<0.0001
Age	18-40 versus 41-50 years	1.794	0.856	3.759	0.1216
Age	51-60 versus 41-50 years	1.013	0.661	1.553	0.9527
Age	61-70 versus 41-50 years	1.147	0.764	1.722	0.5092
Age	71-80 versus 41-50 years	1.518	1.016	2.269	0.0416
Age	81+ versus 41-50 years	2.345	1.562	3.521	<0.0001
Sex	Male versus female	1.287	1.165	1.422	<0.0001
German nationality	No versus yes	1.102	0.930	1.307	0.2614
Living in urban area	No versus yes	1.047	0.955	1.149	0.3262
Employed	Yes versus no	0.732	0.608	0.882	0.0010
State of hardship	Yes versus no	1.442	1.306	1.592	<0.0001
In need of nursing care	Yes versus no	1.511	1.336	1.710	<0.0001
Nursing home resident	Yes versus no	0.698	0.476	1.023	0.0651
DMP CAD	Yes versus no	1.060	0.945	1.190	0.3179
DMP DM	Yes versus no	1.144	0.994	1.316	0.0610
CVD hospitalisation	Yes versus no	1.292	1.158	1.442	<0.0001
Influenza vaccination	No versus Yes	1.126	1.026	1.237	0.0126
Charlson index	Per point	1.019	0.993	1.046	0.1512
NYHA class	Class II versus I	1.404	1.112	1.774	0.0044
NYHA class	Class III versus I	2.821	2.252	3.533	<0.0001
NYHA class	Class IV versus I	4.875	3.808	6.241	<0.0001
NYHA class	No NYHA class versus class I	1.809	1.441	2.271	<0.0001
Diabetes mellitus	Yes versus no	1.251	1.090	1.434	0.0014
Hyperlipidaemia	Yes versus no	0.889	0.803	0.984	0.0229
Renal failure	Yes versus no	1.591	1.420	1.782	<0.0001
COPD	Yes versus no	1.307	1.171	1.458	<0.0001
Pneumonia	Yes versus no	1.052	0.898	1.233	0.5292
Depression	Yes versus no	0.948	0.849	1.060	0.3493
Hypertension	Yes versus no	0.925	0.766	1.116	0.4141
CAD	Yes versus no	1.049	0.928	1.187	0.4456
Atrial fibrillation	Yes versus no	1.860	1.690	2.048	<0.0001
Other arrhythmias	Yes versus no	1.092	0.994	1.199	0.0679
Valvular heart disease	Yes versus no	1.211	1.102	1.331	<0.0001
Myocardial infarction	Yes versus no	1.254	1.113	1.412	0.0002

Supplementary Table S7. Multivariable-adjusted negative binomial regression models for CAD-related hospitalisation in CAD patients enrolled in the cardiology care programme versus control group

CAD, coronary artery disease; CHF, chronic heart failure; COPD, chronic obstructive pulmonary disease; CVD, cardiovascular disease; DMP, disease management programme; DM, diabetes mellitus.

Variables	Interpretation	Rate Ratio	Lower 95% CI	Upper 95% CI	*p*-value
Group	Cardiology care programme versus control group	0.961	0.878	1.052	0.3866
Age	18-40 versus 41-50 years	1.622	0.826	3.183	0.1601
Age	51-60 versus 41-50 years	1.141	0.858	1.517	0.3636
Age	61-70 versus 41-50 years	1.080	0.813	1.433	0.5957
Age	71-80 versus 41-50 years	1.127	0.847	1.500	0.4117
Age	81-90 versus 41-50 years	1.165	0.863	1.573	0.3180
Age	91+ versus 41-50 years	0.968	0.553	1.695	0.9087
Sex	Male versus female	1.082	0.985	1.189	0.1002
German nationality	No versus yes	1.204	1.062	1.365	0.0036
Living in urban area	No versus yes	0.936	0.860	1.020	0.1313
Employed	Yes versus no	0.931	0.803	1.078	0.3369
State of hardship	Yes versus no	1.383	1.260	1.519	<0.0001
In need of nursing care	Yes versus no	0.860	0.741	0.997	0.0458
Nursing home resident	Yes versus no	0.270	0.101	0.719	0.0088
DMP CAD	Yes versus no	1.172	1.067	1.287	0.0009
DMP DM	Yes versus no	0.953	0.836	1.086	0.4702
CVD hospitalisation	Yes versus no	1.613	1.467	1.775	<0.0001
Influenza vaccination	No versus yes	1.041	0.954	1.136	0.3673
Charlson index	Per point	1.035	1.010	1.060	0.0050
Diabetes mellitus	Yes versus no	1.260	1.106	1.434	0.0005
Hyperlipidaemia	Yes versus no	1.273	1.145	1.416	<0.0001
Renal failure	Yes versus no	1.057	0.939	1.189	0.3591
COPD	Yes versus no	1.097	0.984	1.222	0.0945
Pneumonia	Yes versus no	1.143	0.959	1.364	0.1357
Depression	Yes versus no	1.070	0.967	1.184	0.1927
Hypertension	Yes versus no	0.982	0.837	1.152	0.8222
CHF	Yes versus no	0.952	0.863	1.049	0.3186
Atrial fibrillation	Yes versus no	0.844	0.758	0.940	0.0020
Other arrhythmias	Yes versus no	0.876	0.796	0.965	0.0070
Valvular heart disease	Yes versus no	0.926	0.841	1.019	0.1155
Myocardial infarction	Yes versus no	1.536	1.401	1.683	<0.0001
